# Exposure to soluble platinum salts in precious metal refinery workers over a 17-year period

**DOI:** 10.1093/annweh/wxad023

**Published:** 2023-04-27

**Authors:** Lidwien A M Smit, José Jacobs, Juliete da Silva, Dick Heederik, Frits van Rooy, Lützen Portengen, Remko Houba

**Affiliations:** Institute for Risk Assessment Sciences, Utrecht University, Utrecht, The Netherlands; Institute for Risk Assessment Sciences, Utrecht University, Utrecht, The Netherlands; Institute for Risk Assessment Sciences, Utrecht University, Utrecht, The Netherlands; Institute for Risk Assessment Sciences, Utrecht University, Utrecht, The Netherlands; Institute for Risk Assessment Sciences, Utrecht University, Utrecht, The Netherlands; Arbo Unie, Expert Center for Chemical Risk Management, Utrecht, The Netherlands; Institute for Risk Assessment Sciences, Utrecht University, Utrecht, The Netherlands; Institute for Risk Assessment Sciences, Utrecht University, Utrecht, The Netherlands; Netherlands Expertise Center for Occupational Respiratory Disorders, Utrecht, The Netherlands

**Keywords:** chloroplatinate exposure, exposure modelling, job history, low molecular weight allergens, occupational allergy

## Abstract

**Background:**

Occupational exposure to soluble chlorinated platinum (Pt) salts, commonly called chloroplatinates, is a known cause of Pt salt sensitisation (PSS) and occupational asthma. We aimed to model inhalable soluble Pt salts exposure levels based on measurements in precious metal refineries for use in a retrospective cohort study on PSS.

**Methods:**

Five platinum refineries located in the United Kingdom (3 sites), United States, and South Africa provided time weighted average inhalable soluble Pt salts exposure data, measured in 2,982 personal air samples over a 17-year period (2000-2016). We used a Bayesian hierarchical model to estimate geometric mean (GM) exposure levels for each refinery and job title over time.

**Results:**

The GM of measured exposure levels over all facilities was 92 ng/m^3^ with a geometric standard deviation (GSD) of 9.07. Facility-specific GMs ranged from 48 ng/m^3^ (GSD 15.3) to 242 ng/m^3^ (GSD 5.99). Exposure modelling showed that soluble Pt salts exposure levels declined approximately 10% per year in two of the five facilities, but there were no clear time trends in the other facilities. A priori specified exposure groups captured most of the between-jobs differences, which helps to accurately predict exposures for jobs with no measurement data available.

**Conclusions:**

We applied exposure modelling to estimate time, refinery, and job-specific soluble Pt salts exposures. A significant annual decline in exposure levels was observed in two of the five participating facilities. Modelled exposure levels can be linked to individual workers’ job history for exposure–response analysis of PSS in an epidemiological study.

## What’s important about This Paper?

Chloroplatinates, soluble chlorinated platinum salts, are well-known low molecular weight occupational respiratory and skin sensitisers, but evidence on exposure characteristics and exposure–response relations remains scarce. This study analysed inhalable soluble platinum salts exposure levels measured in 2,982 personal air samples, collected in five precious metal refineries over 17 years (2000-2016). There was, approximately, a 10% annual decline in exposure levels in two of the five participating facilities, while a decreasing trend was not observed in the other facilities. Modelled exposure levels can be linked to individual job history information of platinum refinery workers for exposure–response analysis of platinum salt sensitisation.

## Background

Platinum (Pt) is a rare and precious metal that is highly resistant to corrosion, tarnish, and high temperatures (Yam, 2010). The most widespread use of Pt is as a catalyst, in particular in the automobile and petrochemical industry, but Pt also finds important uses in the production of jewellery, ceramic glass, electronics, chemotherapeutic agents, and a variety of medical devices (Yam, 2010; Linde et al., 2017). Along with other Platinum Group Metals (PGMs), Pt is obtained by primary production from mined ore and by secondary production from recycled catalytic converters, electronic equipment, and jewellery (Linde et al., 2017).

Primary and secondary Pt refinery processes involve dissolving of PGM concentrate using acids, followed by precipitation of the metals in the form of complex halogenated precious metal salts. Soluble chlorinated Pt salts, commonly called chloroplatinates, are well-known low molecular weight occupational respiratory and skin sensitisers. Chloroplatinates can act as haptens by combining with endogenous proteins, resulting in potent antigens that can activate a Type I hypersensitivity reaction producing specific IgE (Cleare et al., 1976; Agius et al., 1991).

Occupational exposure to soluble Pt salts occurs especially during the refining process through inhalation or dermal contact (Calverley et al., 1995; Health Council of the Netherlands, 2008; Heederik et al., 2016). Exposure is usually assessed by measuring water-soluble Pt salts as a surrogate for chloroplatinates in the inhalable dust fraction (Shafer et al., 2022). A widely used 8-h time weighted average (TWA) Threshold Limit Value (TLV^®^) for water-soluble Pt compounds of 2,000 ng/m^3^ was established by the American Conference of Governmental Industrial Hygienists in 1963 (Health Council of the Netherlands, 2008). This TLV^®^ was based on limited air sampling data, and was established in absence of quantitative exposure–response evidence. Several studies have reported Pt salt sensitisation (PSS) incidence at exposure levels below the TLV^®^, but evidence on exposure–response relations remains scarce (Merget et al., 2000; Heederik et al., 2015; Linde et al., 2017). In 2008, the Dutch Expert Committee on Occupational Standards (DECOS) recommended a health-based occupational exposure limit (OEL) of 5 ng/m^3^, primarily based on the study by Merget et al. (Merget et al., 2000; Health Council of the Netherlands, 2008).

In a retrospective cohort study in newly hired workers of five precious metal refineries who entered the industry between 2000 and 2010, Heederik et al. (2016) assigned a geometric mean (GM) Pt salts exposure level to each job title. Exposure levels were assumed to be constant for the entire study period. For job titles with six or more measurements, the average exposure level was calculated, while exposure for the other job titles was assigned by combining job titles or ranking job titles on the basis of expert judgement (Heederik et al., 2016). A positive exposure–response relation with PSS was observed at GM soluble Pt salts exposure levels up to 200 ng/m^3^. This study emphasised the need to obtain more evidence on exposure characteristics and the exposure–response relationship, especially at exposure levels in the low ng/m^3^ range. Insight in these aspects can help in developing effective tailormade exposure prevention strategies. Ultimately, more scientific evidence should lead to establishing a health-based OEL to support further reduction of PSS incidence.

In the present study, we analysed inhalable soluble Pt salts exposure levels measured in 2,982 personal air samples, collected in five precious metal refineries over 17 years (2000-2016). At each facility, exposure data were available for 13-15 of these years. The more recent exposure measurements have been taken using improved analytical techniques with lower limits of detection (LOD). We aimed to estimate individual Pt salts exposures by modelling time trend, refinery, and job-specific exposure levels over the 17-year period. Modelled exposure levels can be linked to individual job history information of Pt refinery workers for exposure–response analysis of PSS, which will be reported elsewhere.

## Methods

### Study design

Five platinum refineries, located in the United States (n=1), United Kingdom (n=3), and South Africa (n=1) participated in the study. The refineries provided exposure data routinely collected between January 1, 2000 and December 31, 2016. One was a primary refinery processing only locally produced PGM concentrate, three were secondary refineries, and one was a mixed facility processing both PGM concentrate and PGM-containing materials. All facilities participated in the previous study (2000-2010) (Heederik et al., 2016) and were re-visited between 2016 and 2019 in order to better understand the Pt refining process, tasks, and job titles, and to observe routine exposure measurement procedures.

### Personal exposure measurements

Personal TWA measurements were taken with the sampling head positioned in the breathing zone using a 2 L/min air sampling flow rate, consistent with measuring inhalable dust. Air sampling methods varied by year and facility (Table 1). Soluble Pt salts levels on the loaded filters were extracted and analysed by commercial laboratories or by the companies using analytical methods shown in Table 1. The crude exposure dataset contained 4,923 measurements with information on sampling date, Pt concentration per filter, analytical LOD, measurement duration, sampling volume, flow rate, sampling strategy, sampling type (personal vs. stationary), analysed Pt type (soluble or total), air sampling method, analytical method, facility type, work site and job title. Measurements eligible for the present study were (1) analysed for soluble Pt salts, (2) personal sampling measurements, and (3) the sampling duration lasted between 180 to 660 minutes. Short-term task-based measurements were excluded from the dataset and all measurements therefore represent personal soluble Pt salt exposure during a substantial part of a work shift and were therefore considered representative for a full-shift exposure.

**Table 1.** Overview of air sampling and soluble Pt salts analysis methods and their limits of detection.

Facility	Production Type	Years	Sampling Method	Dust fraction	Analysis Method	LOD (ng)	N
**1**	Secondary	2004, 2005, 2009	MDHS 46/2	Inhalable (IOM sampling head)	GF-AAS	100/1000	12
		2005-2016			ICPMS	10/100	335
		2009-2010			High sensitivity ICPMS	1	44
		2009			Missing	Missing	1
**2**	Secondary	2000, 2002-2009	MDHS 46/2	Inhalable (IOM sampling head)	GF-AAS	10/100/200	46
		2009-2016	MDHS 46/2		ICPMS	10/100/200	290
		2009-2010	MDHS 46/2		High sensitivity ICPMS	1	29
		2016	IPA		ICPMS	10	4
**3**	Primary and secondary	2000-2003, 2005-2009	MDHS 46/2	Inhalable (7-hole sampling head)	ICP-OES	200	322
		2010-2015			ICP-OES ENA011/012/013	200	240
		2012, 2013			Missing	2	49
**4**	Secondary	2001-2010	OSHA ID-130-SG	Total dust (closed-face 37mm cassettes)	NIOSH 7300M ICPMS	100	266
		2010-2015			OSHA121AP2	0.8	463
**5**	Primary	2003-2007	NIOSH 7300	Until 2009 Total dust (closed-face 37mm cassettes)	ICP-AES	380^*^	243
		2008-2009	NIOSH 7300		ICP-OES	380^*^	90
		2010-2015	MDHS 46/2	Inhalable (IOM sampling head)	ICPMS	5	548

LOD, limit of detection (absolute value reported by the facilities or assigned by IRAS experts, which can be considered as a limit of quantification); N, number of measurements (total N=2,982); ICP-AES, inductively coupled plasma atomic emission spectroscopy; ICP-OES, inductively coupled plasma optical emission spectrometry; ICPMS, inductively coupled plasma mass spectrometry; GF-AAS, graphite furnace atomic absorption spectrometry.

^*^Measurements with missing LOD (not provided by the facility). The LOD assigned was based on the collection method LOD established by the method developer.

After eligibility selection, the final dataset contained 2,982 measurements. Reasons to exclude 1,941 measurements were: non-soluble Pt analysis (n=408), stationary sampling (n=50), nonroutine sampling (n=185), missing data, e.g. unknown sampling duration (n=231), sampling duration <180 or >660 minutes (n=311), and duplicates (n=756). The last six years of data collection (2011-2016) contributed 1,434 measurements, corresponding to approximately 48% of the dataset. For the current study, data management and modelling was started from scratch, using all crude datasets originally submitted by each company (2000-2010), (Heederik et al., 2016) supplemented with new information (2010 data not being part of the previous study, and 2011-2016).

The facilities provided information on the analytical LOD value for most of the measurements, which can be considered as limits of quantification (Table 1). For measurements with missing LOD, the LOD of measurements with the same collection and analytical method, taken during the same time period was assigned. If no information from similar measurements was available, the LOD as provided by the collection and analytical method developer was assigned. For exposure modelling purposes, we estimated the LOD (LOD_Sample, ng/m^3^) for each individual measurement with soluble Pt salts concentration below the analytical LOD by dividing the absolute LOD (in ng) on the filter by the sampling volume (m^3^) for that particular measurement.

### Exposure modelling

The dataset of 2,982 exposure measurements was used to estimate individual exposures by modelling exposure for each job title over the entire follow-up period. Exposure modelling was done for two reasons: some job titles have no or very sparse measurement data, which can also be highly variable, while other job titles may have adequate exposure measurement data, but only for certain years of the follow-up period. To estimate job-specific exposures and time trends in exposure levels, we used a hierarchical model with job titles nested within exposure groups based on prior expert knowledge. Similar approaches for occupational exposure assessment have been described before (Peters et al., 2016; Ramachandran, 2019; Sauvé et al., 2020).

**Figure 1.**
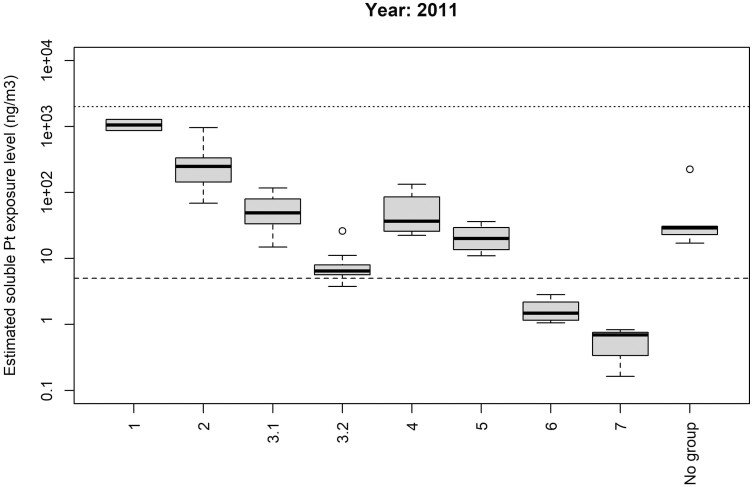
Estimated job-specific soluble Pt exposure levels (ng/m^3^) by exposure group in the reference year 2011. Dotted line: TLV® 2000 ng/m^3^. Dashed line: DECOS health-based OEL 5 ng/m^3^. **Group 1:** Continuous exposure during regular work shifts; geometric mean (GM) >1000 ng/m^3^. Group 1 will be excluded from subsequent exposure–response analysis because there is no involvement of chlorine here and therefore no potential for generation of chloroplatinates. **Group 2:** Continuous exposure during regular work shifts; GM 100-1000 ng/m^3^_._**Group 3.1:** Continuous exposure during regular work shifts; GM <100 ng/m^3.^ Workers in this group are all production workers with likely soluble Pt salt exposure in regular Pt production areas. **Group 3.2:** Continuous exposure during regular work shifts; GM <100 ng/m^3^. Workers in this group have been separated from group 3.1 as they were not working in regular Pt production areas. Either they are laboratory workers or they work in production areas with low Pt salt exposure potential but still Pt areas. **Group 4:** Variable exposure, mainly maintenance workers. **Group 5:** Occasional exposure; not full-time in the plant but occasionally entering the production area as part of their job. **Group 6:** Production workers in non-Pt areas. **Group 7:** Non-exposed; full office-workers; non-production workers never going into the plant. **No group:** Exposure codes that did not fit in any of the groups above and were also not similar to each other.

According to expert evaluation of the different facilities, the refinery processes and procedures and resulting exposure profiles for workers were substantially different. Therefore, a facility-specific job classification (exposure codes) was developed for each facility. To improve exposure estimation for jobs with no or few measurements available (e.g. some of the laboratory or office job titles), all exposure codes were assigned to one of eight different exposure groups. The exposure groups are not facility-specific, and were defined based on knowledge of the process and tasks, and the (expected) average exposure levels in relation to other job titles with sufficient exposure information, and whether exposure was continuous, variable, occasional, or absent (exposure groups and the exposure codes belonging to the groups are shown in Tables S1 and S2, Supplementary material, and Figure 1).

The distribution of soluble Pt salt concentrations was right-skewed and measurements were therefore log-transformed before modelling. Because 41% of the measurements were below the LOD, we used a Bayesian hierarchical model that allowed for left-censoring to estimate facility-specific and job-specific GMs (GM) for soluble Pt salt exposure over time. The left-censoring was accommodated by considering a censored observation as missing data known to be below the measurement-specific LOD (LOD_sample) and using a cumulative (normal) distribution function to model its contribution to the likelihood.

Facility, exposure group, and exposure code information were used in a hierarchical model, with measurements (level 1) nested in exposure codes (level 2) and exposure codes nested in exposure groups (level 3). Assuming a linear exposure time trend, the (level 1) model for the observed (log-transformed) measurement data is:


$$\begin{array}{l} Y_{ijk}∼N(E(Y_{ijk}),\sigma_{i}) \end{array}$$

$$\begin{array}{l} E\left(Y_{ijk}\right)=\beta_{0,ij}+\beta_{t,ij}*T_{ijk} \end{array}$$

for the k^th^ measurement on exposure code *j* in facility *i* at time *T*_ijk_. The *year* variable was median centred before exposure modelling, thus intercepts relate to estimated exposure levels for the year 2011.

The (level 2) model for the coefficients of the level 1 model is:


$$\begin{array}{l} \beta_{0,ij}=\delta_{0,i}+\varphi_{0,ij} \end{array}$$

$$\begin{array}{l} \beta_{t,ij}=\delta_{t,i}+\varphi_{t,ij} \end{array}$$

In this model, parameters $\delta_{0,i}$ and $\delta_{t,i}$ are the overall (average) facility-specific intercept and slope (time trend) for facility *i*, while the $\varphi_{0,ij}$ and $\varphi_{t,ij}$ are exposure code-specific intercepts and slopes respectively.

The (level 3) model for these exposure code-specific parameters is:


$$\begin{array}{l} \varphi_{0,ij}=\eta_{0,g_{j}}+\gamma_{0,ij} \end{array}$$

$$\begin{array}{l} \varphi_{t,ij}=\eta_{t,g_{j}}+\gamma_{t,ij} \end{array}$$

where $\eta_{0,g_{j}}$ and $\eta_{t,g_{j}}$ respectively $\gamma_{0,ij}$ and $\gamma_{t,ij}$ are modelled as exposure group and exposure code-specific random effects, assumed to follow separate multivariate normal (MVN) distributions, with $\sum_{\eta }$ and $\;\sum_{\gamma }$ the corresponding random effect (co)variances:


$$\begin{array}{l} \left[\begin{array}{l} \gamma_{0,ij}\\ \gamma_{t,ij} \end{array}\right]∼MVN\left(\left[\begin{array}{l} 0\\ 0 \end{array}\right],\;\sum_{\gamma }\right) \end{array}$$

$$\begin{array}{l} \left[\begin{array}{l} \eta_{0,g_{j}}\\ \eta_{t,g_{j}} \end{array}\right]∼MVN\left(\left[\begin{array}{l} 0\\ 0 \end{array}\right],\;\sum_{\eta }\right) \end{array}$$

Here *g*_*j*_ indicates the exposure group (group 1, 2, 3.1, 3.2, 4, 5, 6, 7, or no group, *see*Table S2 and Figure 1) in which the exposure code is nested (*see*Table S1).

Different model structures were explored by eliminating selected exposure code-level and/or exposure-group-level coefficients from this general model (effectively assuming these were 0), or by modelling the group-level coefficients as fixed effects. Bayes factors were used to assess (relative) model fit and inform final model choice (Kass and Raftery, 1995).

The model was implemented using the *brms* package in R, which is a convenient wrapper for the efficient Markov-Chain Monte-Carlo (MCMC) algorithms that are implemented in the Stan language (Bürkner, 2017). Exposure estimates were obtained from 4 chains for 2,000 MCMC iterations per chain, after a burn-in of 1,000 iterations. Both convergence and the effective number of samples were checked and were considered adequate. The *adapt_delta* parameter of the algorithm was modified from its default value to 0.9. This makes the sampler take smaller steps, making it less likely to miss small areas with high posterior probability, but also increasing the number of iterations needed to fully explore the posterior distribution.

Samples from the conditional posterior predictive distribution of the model were used to estimate GM soluble Pt salt exposures for each job title for each year between 2000 and 2016, and to impute measurements below the LOD in order to report descriptive statistics of measured exposure levels per facility. Point estimates were calculated as the mean value across 8,000 iterations, and empirical 2.5% and 97.5% quantiles were used to estimate 95% credible intervals (95%CIs) for these estimates. For job titles that had no measurement data available, point estimates were based on the estimated GM for the facility and the exposure group to which the job title was assigned, with the 95%CIs accounting for the between-job variation in exposure levels.

## Results

### Measured soluble Pt salts exposure levels (2000-2016)

In 41% of 2,982 available personal exposure measurements, levels of soluble Pt salts were non-detectable (Table 2; %ND per year in Table S3, Supplementary material). Up to 2011, overall annual percentages of non-detects ranged between 25% and 81%. Since 2012, the maximum overall annual percentage of non-detects was 27%. In facility 3, 63% of all samples had levels below the LOD, followed by facility 5 with 44% of samples below the LOD, while refinery 2 presented the lowest percentage of measurements below the LOD (17%).

**Table 2.** Overview of soluble Pt salts levels (ng/m^3^) from 2,982 personal measurements in five facilities between 2000 and 2016.

Facility	N	ND	AM	GM	GSD	P5	P95
**1**	392	89 (23%)	929	176	5.67	10.0	3,963
**2**	369	62 (17%)	1,715	242	5.99	12.6	5,492
**3**	611	382 (63%)	489	152	3.51	31.0	1,484
**4**	729	286 (39%)	326	56	8.73	0.80	1,420
**5**	881	390 (44%)	1,508	48	15.3	0.85	4,042
**All**	2,982	1,209 (41%)	960	92	9.07	1.28	2,732

N, number of measurements; ND, non-detectable measurements; AM, arithmetic mean (ng/m^3^); GM, geometric mean (ng/m^3^); GSD, geometric standard deviation; P5, 5^th^ percentile (ng/m^3^); P95, 95^th^ percentile (ng/m^3^).

The GM of measured personal exposure levels over all facilities was 92 ng/m^3^ with a geometric standard deviation (GSD) of 9.07 (Table 2). When analysed by refinery, facility 5 had the lowest GM exposure (48 ng/m^3^) but the highest variability (GSD 15.3). The highest average exposure level was measured in facility 2 (GM 242 ng/m^3^, GSD 5.99), where the highest 95-percentile of exposure levels was found as well (5,492 ng/m^3^).

Table S1 shows an overview of the 2,982 measured soluble Pt salts concentrations stratified by exposure code (facility-specific job title). Exposure distributions within exposure codes were typically large (82% had GSD greater than 3) and often were extremely large (22% had a GSD greater than 10). In facility 1, the highest GMs were found in exposure code 103 (Evaluation – high grade area) with a GM of 500 ng/m^3^ (GSD 6.05) and exposure code 112 (Other) with a GM of 411 ng/m^3^ (GSD 2.26). In facility 2, exposure code 206 (Products – Lab 8-10) had the highest GM level of 1,076 ng/m^3^. The GSD of 16.6, and the fact that 8 out of 16 measurements were below the LOD also indicated high variability. In facility 3 the highest GM of 498 ng/m^3^ (GSD 4.32) belonged to exposure code 301 (Process operators after HCl/Cl_2_ leach involved in Pt salt digging). Exposure code 411 (Chemicals Unspecified) had the highest average exposure in facility 4 with a GM of 171 ng/m^3^ (GSD 3.13), which was substantially lower than high exposed job titles in the other facilities. In facility 5, exposure code 501 (Evaluation – process operator) had the highest GM of 814 ng/m^3^ (GSD 8.53), with only 4 out of 63 measurements below the LOD. However, there is no involvement of chlorine in the work area for exposure codes 501-503, because it receives final concentrate from the base metal refinery, and therefore no potential for generation of chloroplatinates.

Measured soluble Pt salts concentrations stratified by exposure code in the first (2000-2010) and second (2011-2016) part of the study are shown in Supplementary Table S4. For most job titles, GM exposure levels were higher in the first than in the second part of the study, with a few exceptions, notably in facility 2 where most job titles showed an increase in GM exposure.

### Soluble Pt salts exposure model

Based on Bayes factors, a model with facility-specific intercepts, time trends and variances, exposure-group specific intercepts, and random job-specific intercepts and time trends was selected as the best model for predicting job- and time-specific soluble Pt salts exposures (Table 3).

**Table 3.** Exploration of different model structures. Bayes factors were used to assess (relative) model fit and inform final model choice (bold type).

Facility-specific model	Random effects		ΔMLL	BF
	Exposure groups	Exposure codes		
$\delta_{0,i}$ , $\delta_{t,i}$	$\eta_{0,g_{j}}$ , $\eta_{t,g_{j}}$	$\gamma_{0,ij}$ , $\gamma_{t,ij}$	-58.1	5.7E-26
$\delta_{0,i}$ , $\delta_{t,i}$, $\sigma_{i}$	$\eta_{0,g_{j}}$ , $\eta_{t,g_{j}}$	$\gamma_{0,ij}$ , $\gamma_{t,ij}$	0.0	1
$\delta_{0,i}$ , $\delta_{t,i}$, $\sigma_{i}$	$\eta_{0,g_{j}}$	$\gamma_{0,ij}$ , $\gamma_{t,ij}$	**4.7**	**112**
$\delta_{0,i}$ , $\delta_{t,i}$, $\sigma_{i}$	-	$\gamma_{0,ij}$ , $\gamma_{t,ij}$	-30.1	8.4E-14
$\delta_{0,i}$ , $\delta_{t,i}$, $\sigma_{i}$	-	$\gamma_{0,ij}$	-34.5	1.1E-15
$\delta_{0,i}$ , $\delta_{t,i}$, $\sigma_{i}$	$\eta_{0,g_{j}}$ , $\eta_{t,g_{j}}$	-	-13.7	1.1E-06
$\delta_{0,i}$ , $\delta_{t,i}$, $\sigma_{i}$	$\eta_{0,g_{j}}$	-	-13.5	1.3E-06
$\delta_{0,i}$ , $\delta_{t,i}$, $\sigma_{i}$	-	-	-331.8	8.3E-145

MLL, maximum likelihood; BF, Bayes factors; $\delta_{0,i}$, facility-specific intercept for facility *i*; $\delta_{t,i}$, facility-specific slope (time trend) for facility *i*; $\sigma_{i}$, facility-specific residual standard deviation for facility *i*; $\eta_{0,g_{j}}$, random exposure-group specific intercept for the group to which exposure code *j* belongs; $\eta_{t,g_{j}}\;$, random exposure-group specific slope (time trend) for the group to which exposure code *j* belongs; $\gamma_{0,ij}$, random exposure code-specific intercept for exposure code *j* in facility *i*; $\gamma_{t,ij}$, random exposure code-specific slope (time trend) for exposure code *j* in facility *i*.

Parameter estimates for the selected model are provided in Table 4. Exposures declined significantly over time in facilities 3 and 5 with decreases of approximately 10% per year on average (GM ratio of 0.90 and 0.89, respectively), but there was little evidence of similar trends in the other facilities.

**Table 4.** Model parameters of the final chloroplatinate exposure model.

Facility-specific effects			
Facility	GM	GMR	$\sigma_{i}^{2}$
1	54.1 [6.8, 419.9]	0.97 [0.89, 1.06]	1.60 [1.48, 1.75]
2	49.9 [5.5, 395.4]	1.08 [0.97, 1.23]	1.63 [1.51, 1.79]
3	57.4 [7.0, 424.1]	0.89 [0.83, 0.94]	1.45 [1.32, 1.60]
4	24.0 [2.9, 192.5]	1.04 [0.96, 1.13]	2.36 [2.20, 2.53]
5	16.4 [2.0, 127.7]	0.90 [0.82, 0.98]	2.46 [2.29, 2.64]
**Random effects**			
**Exposure groups**			
var$\left(\eta_{0,g_{j}}\right)$	7.51 [2.25, 26.11]		
**Exposure codes**			
$var(\gamma_{0,ij})$	0.31 [0.14, 0.59]		
$var(\gamma_{t,ij})$	0.010 [0.004, 0.020]		
$cor(\gamma_{0,ij},\gamma_{t,ij})$	0.38 [-0.27, 0.82]		

GM: the facility-specific GM chloroplatinate exposure level (for the reference year 2011) is obtained by exponentiating $\delta_{0,i}$, the intercept for facility *i*; the GMR (geometric mean ratio) is obtained by exponentiating $\delta_{t,i}$, the facility-specific slope (time trend) for facility *i*; $\sigma_{i}^{2}$, residual variance for facility *i*; $var(\eta_{0,g_{j}})$, variance of random exposure-group specific intercept for the group to which exposure code *j* belongs; $var(\gamma_{0,ij})$, variance of random exposure code-specific intercept for exposure code *j* in facility *i*; $var(\gamma_{t,ij})$, variance of random exposure code-specific slope (time trend) for exposure code *j* in facility *i*; $cor(\gamma_{0,ij},\gamma_{t,ij})$ correlation between $\gamma_{0,ij}$ and $\gamma_{t,ij}$.

**Figure 2.**
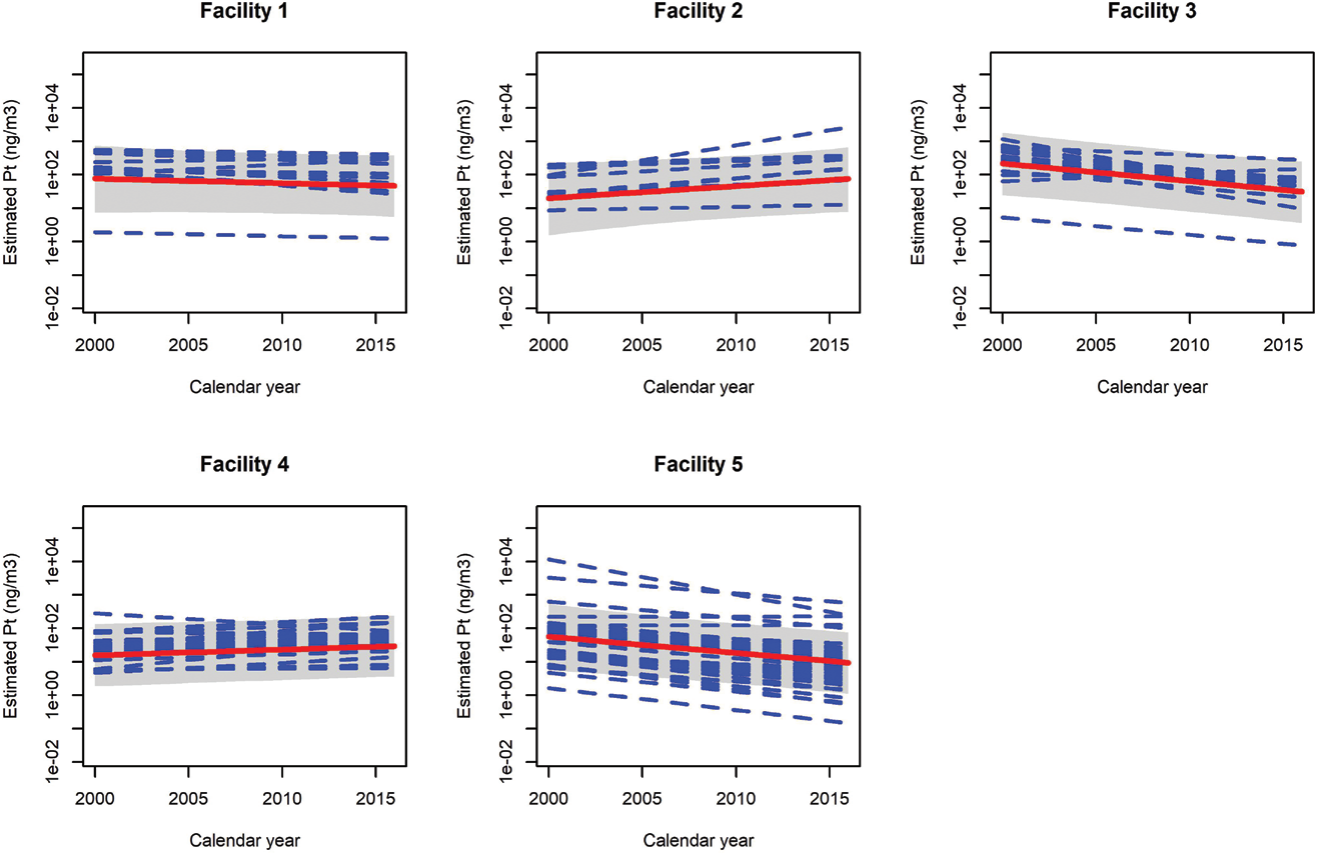
Facility-specific overall trends in estimated GM chloroplatinate exposure levels (ng/m^3^) (linear trend line) with 95% credible interval (area), and job-specific trends overlaid (dashed linear trend lines). Please note that the estimated GM can differ from those presented in Table 1. This is because of the lognormal distribution and random effects structure. Part of the variation between measurements is now captured by the jobs (exposure codes), which means that a GM accounting for between-job variation is lower than one that doesn’t.

Figure 1 presents boxplots of estimated job-specific exposure levels by exposure groups in the reference year 2011. Estimated exposure levels per group show good agreement with a priori specified exposure ranking. Figure 2 shows facility-specific overall trends with job-specific trends overlaid. The relatively high estimated between-jobs variance in trends suggests significant heterogeneity in trends between jobs within a facility. Trends for 10 jobs do not track with the facility-wide trend (indicated in Table S1 and S4), especially in facility 4 where five job-specific trends show a decrease in exposure levels while the overall trend shows a slight (nonsignificant) increase. The large between-exposure-groups variance in intercepts compared to that for jobs (see Table 4, 7.51 versus 0.31, an approximately 24-fold difference) confirms that these a priori specified groups (Figure 1) were able to capture most of the between-jobs differences, which may be important for predicting exposures for jobs that have no measurement data available.

## Discussion

We analysed data on soluble Pt salts concentrations in 2,982 personal air samples from precious metal refinery workers from five different facilities taken over a 17-year period. Exposure levels declined significantly over the study period in two of the five facilities, with an average decrease of approximately 10% per year, but no similar trends were observed in the other facilities. Approximately 6% of all 2,982 personal exposure measurements taken between 2000 and 2016 exceeded the TLV^®^ of 2,000 ng/m^3^ and 63% of all measurements exceeded the health-based OEL of 5 ng/m^3^ recommended by DECOS (Health Council of the Netherlands, 2008). TLV^®^ exceedances were found across 36% of all exposure codes with at least one personal exposure measurement available.

Exposure–response curves were previously estimated using data from the 11-year follow-up (2000-2010) of the present study (Heederik et al., 2016). In those analyses, soluble Pt salts exposure concentrations were more frequently measured using techniques with a relatively high LOD as shown in Table 1, and exposure levels were imputed to estimate average exposure levels for many relatively low exposed job titles. The current study used almost double the number of personal exposure measurements, including more recently collected exposure data with analytical techniques with a lower LOD. By applying more sophisticated exposure modelling including time trends in exposure levels and a priori specified exposure groups, we estimated job-specific and year-specific exposure levels that can be assigned to individual workers in the cohort, in order to update exposure–response relationships for PSS.

In general, it is assumed that decreases in chloroplatinate concentrations over the last decades in Pt processing industries have gradually led to a much lower PSS incidence (Venables et al., 1989; Calverley et al., 1995; Niezborala and Garnier, 1996; Merget et al., 2000; Heederik et al., 2016). However, only few studies reported personal Pt salts exposure levels in detail. Linde et al. measured respiratory exposure to soluble Pt in forty precious metal refinery workers over two consecutive work shifts at two South African refineries in 2015 and 2016 (Linde et al., 2021b). They reported a GM of 345 ng/m^3^, but also a large range of exposure levels across job groups, with highest levels in workers handling PGM concentrate. In a catalyst production plant in Italy, the highest soluble and total Pt levels in stationary and personal air samples were measured in the department of the plant in which supports are coated with acid metal solutions (Petrucci et al., 2005). In the administration department of the plant, Pt levels were much lower than in the processing areas, but much higher compared to levels measured in external control areas (Petrucci et al., 2005). Maynard et al. collected short-term and long-term air samples at three Pt processing sites. In general, soluble Pt salts exposure levels were below the TLV^®^ of 2,000 ng/m^3^ while recent cases of PSS had been identified at all three sites (Maynard et al., 1997).

In the current study, data collection was not specifically designed for a scientific study, as we used exposure data for total soluble Pt routinely collected by occupational hygienists from each facility without a common study protocol or quality controls. Using clear inclusion criteria in combination with exposure modelling, we have made the best use of the available company exposure data optimising representation of full-shift soluble Pt salt exposure. Although the more recent data included a lower percentage of non-detects, companies should be encouraged to use analytical techniques with a lower LOD to obtain more exposure data at the lower ng/m^3^ range. Because of analytical practicality, total soluble Pt is generally measured as a proxy for potential exposure to inhalable chloroplatinates (Shafer et al. 2022). Although soluble halogenated platinum compounds are most relevant to PSS, other soluble Pt compounds may harbour sensitisation potential as well, so modelling exposure to total soluble Pt can be regarded as a precautionary approach for future epidemiological research (Shafer et al. 2022). For some of the job titles no or only a limited number of measurements were available, which will potentially result in exposure misclassification. Exposure modelling implies that an individual worker can be assigned an exposure level that differs from the individual measurement data, in particular for jobs for which relatively few measurements are variable, or taken in very different time periods. The a priori specified groups in the exposure model captured most of the between-jobs differences, which allows predicting exposures for jobs with no measurement data available. Most job-specific trends were estimated rather imprecisely, and may contain artefacts due to measurement data being only patchily available for most jobs. Note that the sparse measurement data and uneven sampling moments for many jobs would make reliable estimation of non-linear time trends challenging, but the range of measurement years spanned 17 years, which seems narrow enough to allow using a linear time trend, accepting that in reality changes in exposure level typically occur as step-changes from new engineering controls. However, our study was not designed to study the impact of control measures or other determinants associated with decreasing or increasing trends in exposure levels for facilities or specific job titles.

It has been argued that PSS may arise from short-term very high exposures, although there is little evidence to substantiate this claim (Maynard et al., 1997). The exposure model described here estimates average exposure levels representative of a full-shift exposure. Accidental or occasional peak concentrations may not be adequately captured in the average concentrations that are based on personal exposure measurements during at least three hours because these unplanned and irregularly occurring events are seldom covered by such a sampling time window. It should be realised that evaluation of the role of peak exposures requires high resolution, long duration and highly unpractical and expensive sampling strategies. Real-time exposure assessment is not an option as soluble Pt salts concentrations cannot be detected by sensor techniques. The limited number of (a priori excluded) short-term samples were not deemed suitable for further analysis. Peak concentrations and average exposure within job titles are expected to be highly correlated as shown in some other industries thus making average exposure levels a practical and feasible exposure proxy assuming that peak exposures do play an important biological role (Smit et al., 2006; Meijster et al., 2008; Checkoway et al., 2019; Virji and Kurth, 2021).

While inhalation is undoubtedly the major route of exposure involved in PSS development, dermal contact may also contribute to relevant Pt salts exposures. *In vitro* experiments indicated that Pt salts permeated through intact skin, and that permeation through African skin was significantly higher than through Caucasian skin (Franken et al., 2015). In mouse models, dermal treatment with various halogenated Pt salts resulted not only in sensitisation, but also triggered changes in lung function following respiratory challenge with Pt salts (Lehmann and Williams, 2018). However, it is still uncertain whether dermal exposure under working conditions contributes to respiratory sensitisation in exposed workers. Recent studies have shown moderate to strong correlation between respiratory and dermal exposure levels to soluble Pt in precious metal refineries, and both skin and respiratory exposure were correlated with increased urinary Pt excretion (Linde et al., 2018, 2021b). Because exposure routes occurred simultaneously, the impact of each route could not be studied in isolation. Another recent study showed that the use of disposable coveralls and rubber gloves, along with strict usage procedures, proved effective in reducing skin exposure to soluble Pt, while urinary Pt excretion did not differ significantly from workers who did not wear effective personal protective equipment (PPE) (Linde et al., 2021a). In the present study, only airborne Pt was quantified, but we can assume that dermal exposures co-occurred with respiratory exposure. Dedicated research is required to further understand the role of dermal exposure. Furthermore, it has been suggested that control measures aimed at controlling exposures via both routes can be combined in specific work areas to reduce overall exposure (Linde et al*.*, 2021b). Data on the individual use of PPE was not systematically collected. If higher exposed workers are compliant in using PPE, and adequately control their inhalation exposure by correctly using effective PPE, their true exposure will be lower than the exposure estimated in the current study.

### Conclusion

In conclusion, we applied exposure modelling to estimate time, refinery, and job-specific soluble Pt salts exposures using almost 3,000 personal air samples collected over a 17-year period. We found an approximately 10% annual decline in soluble Pt salts exposure levels in two of the five participating facilities, while a decreasing trend in concentrations was not observed in the other facilities. Modelled exposure levels linked to exposed workers’ job history can be used in epidemiological studies investigating exposure–response relationships with PSS.

## Supplementary Material

wxad023_suppl_Supplementary_Material

## Data Availability

The data underlying this article were provided by participating companies by permission. Data may be shared on reasonable request to the corresponding author with permission of participating companies.
