# Supplementary material for: Exposure to soluble platinum salts in precious metal refinery workers over a 17-year period
Source: Ann Work Expo Health. 2023 Apr 27;67(6):720–30. doi: 10.1093/annweh/wxad023 (PMC10324643; doi:10.1093/annweh/wxad023)
Supplement: wxad023_suppl_Supplementary_Material [file wxad023_suppl_supplementary_material.docx]

**Supplementary Tables**

**Exposure to soluble platinum salts in precious metal refinery workers over a 17-year period**

Lidwien A.M. Smit, José Jacobs, Juliete da Silva, Dick Heederik, Frits van Rooy, Lützen Portengen, Remko Houba

**Table S1.** Overview of personal exposure measurements of soluble Pt salts (ng/m^3^) for each combination of facility and job title (exposure codes) during the full study period between 2000 and 2016.

| **Facility** | **Exposure Code** | **Job title** | **N** | **ND** | **AM** | **GM** | **GSD** | **Min** | **Max** |
| --- | --- | --- | --- | --- | --- | --- | --- | --- | --- |
| 1 | 101 | Evaluation - incineration | 8 | 4 | 806 | 295 | 5.56 | 52 | 2,200 |
|  | 102 | Evaluation - sampling | 78 | 11 | 1,233 | 384 | 4.65 | 30 | 12,000 |
|  | 103 | Evaluation - high grade area | 16 | 0 | 5,335 | 500 | 6.05 | 51 | 75,000 |
|  | 104 | Evaluation - melting/meltshop | 21 | 8 | 413 | 78 | 5.92 | 8 | 3,800 |
|  | 105 | Smelting | 125 | 36 | 803 | 133 | 5.49 | 5 | 16,988 |
|  | 106^†^ | Chemicals - chlorination & Fe bullion | 84 | 12 | 681 | 299 | 3.32 | 10 | 11,000 |
|  | 107 | Chemicals – BSP and SNC | 8 | 0 | 172 | 118 | 2.73 | 30 | 400 |
|  | 108 | Chemicals - Leach & effluent | 21 | 8 | 71 | 39 | 2.85 | 6 | 600 |
|  | 109 | Maintenance workers | 12 | 5 | 35 | 25 | 2.74 | 5 | 63 |
|  | 110 | Laboratory workers | 0 |  |  |  |  |  |  |
|  | 111 | Full office workers | 6 | 5 | 4 | 2 | 2.62 | 0.7 | 13 |
|  | 112^†^ | Other | 13 | 0 | 525 | 411 | 2.26 | 63 | 1,250 |
|  |  |  |  |  |  |  |  |  |  |
| 2 | 201 | Refinery - Process operators | 255 | 28 | 1,085 | 339 | 4.61 | 5 | 18,513 |
|  | 202 | Refinery - Maintenance workers | 39 | 5 | 700 | 128 | 5.07 | 6 | 16,964 |
|  | 203 | Refinery - Lab & development staff | 20 | 14 | 24 | 12 | 2.92 | 2 | 200 |
|  | 204 | Products - Homcat | 11 | 1 | 373 | 272 | 2.38 | 54 | 1,183 |
|  | 205 | Products – Procat 1 | 15 | 3 | 540 | 121 | 4.09 | 20 | 5,560 |
|  | 206 | Products – Labs 8-10 | 16 | 8 | 19,625 | 1,076 | 16.6 | 56 | 193,375 |
|  | 207 | Products – PU11 | 9 | 0 | 230 | 116 | 3.89 | 8 | 1,050 |
|  | 208 | Products – dispensing | 4 | 3 | 46 | 25 | 3.89 | 5 | 125 |
|  | 209 | Effluent Value recovery plant | 0 |  |  |  |  |  |  |
|  | 210 | Full office workers | 0 |  |  |  |  |  |  |
|  | 211 | Other | 0 |  |  |  |  |  |  |
|  |  |  |  |  |  |  |  |  |  |
| 3 | 301 | Process operators after HCl/Cl_2_ leach involved in Pt salt digging | 28 | 11 | 2,248 | 498 | 4.32 | 64 | 33,580 |
|  | 302 | Process operators after HCl/Cl_2_ leach others | 58 | 30 | 393 | 179 | 3.52 | 11 | 2,171 |
|  | 303 | Process operators after nitric acid leach before HCl/Cl_2_ leach | 91 | 50 | 341 | 185 | 3.01 | 33 | 1,508 |
|  | 304 | Ignition area/heat treatment | 25 | 17 | 265 | 100 | 3.81 | 11 | 1,471 |
|  | 305^†^ | Toll sampling area | 62 | 45 | 258 | 115 | 3.29 | 2 | 4,010 |
|  | 306 | Furnace room/lead smelt | 82 | 63 | 381 | 128 | 3.11 | 14 | 8,605 |
|  | 307 | Effluent treatment | 32 | 20 | 263 | 119 | 3.29 | 11 | 1,538 |
|  | 308 | Maintenance workers – fitters | 87 | 55 | 662 | 148 | 3.27 | 11 | 26,083 |
| *Continued on next page* | | | | | | | | | |

**Table S1.** Continued from previous page

| **Facility** | **Exposure Code** | **Job title** | **N** | **ND** | **AM** | **GM** | **GSD** | **Min** | **Max** |
| --- | --- | --- | --- | --- | --- | --- | --- | --- | --- |
| 3 | 309 | Maintenance workers – electrical | 24 | 13 | 579 | 201 | 3.96 | 31 | 3,572 |
|  | 310 | Process control chemists | 36 | 27 | 173 | 95 | 2.84 | 11 | 880 |
|  | 311 | R&D | 20 | 12 | 228 | 157 | 2.34 | 45 | 717 |
|  | 312 | Analytical department | 18 | 13 | 253 | 146 | 2.86 | 38 | 711 |
|  | 313 | Store people | 6 | 3 | 1,034 | 357 | 5.83 | 44 | 2,759 |
|  | 314 | Packing & Dispatch | 1 | 0 |  |  |  | 1,336 | 1,336 |
|  | 315 | Security personnel | 2 | 2 | 62 | 59 | 1.49 | 45 | 79 |
|  | 317 | Engineers designers | 5 | 3 | 780 | 132 | 6.89 | 34 | 3,636 |
|  | 318 | Full office workers | 8 | 5 | 193 | 142 | 2.34 | 40 | 500 |
|  | 319 | Other | 26 | 13 | 747 | 147 | 5.76 | 14 | 4,085 |
|  |  |  |  |  |  |  |  |  |  |
| 4 | 401 | Refinery Foots operator | 18 | 11 | 71 | 33 | 4.22 | 0.9 | 310 |
|  | 402 | Refinery Rh operator | 15 | 6 | 86 | 43 | 3.12 | 7 | 450 |
|  | 403 | Refinery Pd operator | 22 | 11 | 77 | 51 | 2.63 | 7 | 280 |
|  | 404 | Refinery A/R operator | 20 | 10 | 96 | 52 | 3.39 | 7 | 280 |
|  | 405 | Refinery Pt operator | 32 | 20 | 83 | 41 | 3.53 | 4 | 388 |
|  | 406 | Refinery Extraction operator | 35 | 20 | 154 | 69 | 3.83 | 3 | 846 |
|  | 407 | Refinery Unspecified | 0 |  |  |  |  |  |  |
|  | 408 | Chemicals Mezzanine operator | 43 | 11 | 490 | 113 | 12.6 | 0.45 | 5,421 |
|  | 409^†^ | Chemicals Oven room operator | 22 | 10 | 511 | 40 | 22.5 | 0.43 | 2,960 |
|  | 410 | Chemicals main floor operator | 68 | 17 | 676 | 130 | 12.0 | 0.33 | 7,955 |
|  | 411 | Chemicals Unspecified | 16 | 4 | 284 | 171 | 3.13 | 18 | 1,000 |
|  | 412 | Material preparation Ignition operator | 61 | 27 | 359 | 75 | 6.76 | 0.64 | 4,230 |
|  | 413 | Material preparation Main eq operator | 43 | 20 | 331 | 90 | 6.80 | 0.83 | 1,770 |
|  | 414^†^ | Material preparation SR40 operator | 29 | 6 | 988 | 159 | 12.2 | 0.65 | 10,436 |
|  | 415^†^ | Material preparation Ball mill operator | 75 | 26 | 460 | 97 | 8.21 | 0.52 | 4,800 |
|  | 416 | Material preparation Melt room operator | 40 | 17 | 99 | 36 | 5.76 | 0.44 | 730 |
|  | 417^†^ | Material preparation Unspecified | 19 | 9 | 168 | 49 | 7.08 | 0.44 | 1,336 |
|  | 418^†^ | Catalysts Fuel cell operator | 5 | 2 | 92 | 15 | 19.9 | 0.32 | 298 |
|  | 419 | Catalysts Disk area operator | 20 | 8 | 55 | 20 | 8.28 | 0.29 | 160 |
|  | 420 | Catalysts Carbon operator | 34 | 14 | 224 | 27 | 13.2 | 0.59 | 1,900 |
|  | 421 | Catalysts Particulate operator | 15 | 6 | 1,095 | 144 | 5.89 | 20 | 12,860 |
|  | 422 | Catalysts Oven room operator | 3 | 2 | 79 | 18 | 17.4 | 0.76 | 200 |
|  | 423 | Catalysts Unspecified | 1 | 1 |  |  |  | 0.61 | 0.61 |
|  | 424 | Vault Shipper | 4 | 1 | 95 | 17 | 23.1 | 0.25 | 190 |
| *Continued on next page* | | | | | | | | | |

**Table S1.** Continued from previous page

| **Facility** | **Exposure Code** | **Job title** | **N** | **ND** | **AM** | **GM** | **GSD** | **Min** | **Max** |
| --- | --- | --- | --- | --- | --- | --- | --- | --- | --- |
| 4 | 425 | Effluent operator | 12 | 6 | 33 | 17 | 4.31 | 1.3 | 78 |
|  | 426 | Warehouse Forklift driver | 25 | 11 | 173 | 37 | 10.5 | 0.42 | 840 |
|  | 427 | Lab worker Analytical lab (A-lab) | 33 | 5 | 24 | 6 | 4.37 | 0.44 | 460 |
|  | 428 | Lab worker Chemicals development lab | 2 | 1 | 10 | 3 | 14.2 | 0.47 | 20 |
|  | 429 | Lab worker Refining development lab | 6 | 0 | 118 | 63 | 3.31 | 17 | 410 |
|  | 431 | Maintenance worker Mechanics | 10 | 3 | 208 | 34 | 14.5 | 0.63 | 1,000 |
|  | 432 | Maintenance worker Electricians | 0 |  |  |  |  |  |  |
|  | 434 | Maintenance worker Cleaning or janitor maintenance | 0 |  |  |  |  |  |  |
|  | 435 | Maintenance worker Boilermakers | 1 | 1 |  |  |  | 0.59 | 0.59 |
|  | 437 | Maintenance worker Unspecified | 0 |  |  |  |  |  |  |
|  | 438 | Quality people | 0 |  |  |  |  |  |  |
|  | 439 | Security | 0 |  |  |  |  |  |  |
|  | 440 | Managers | 0 |  |  |  |  |  |  |
|  | 441 | Process engineers (coded as engineers) | 0 |  |  |  |  |  |  |
|  | 442 | Health and Safety people | 0 |  |  |  |  |  |  |
|  | 444 | Shipping and receiving (end products of chemicals department) | 0 |  |  |  |  |  |  |
|  |  |  |  |  |  |  |  |  |  |
| 5 | 501^*^ | Area 245/evaluation - process operator | 63 | 4 | 7,257 | 814 | 8.53 | 4 | 142,000 |
|  | 502^*^ | Area 245/evaluation - process coordinator | 22 | 3 | 5,235 | 351 | 9.50 | 2 | 71,190 |
|  | 503^*^ | Area 245/evaluation - other job title | 1 | 0 |  |  |  | 2,450 | 2,450 |
|  | 504 | Bay 1 (any job title) | 8 | 5 | 121 | 5 | 11.0 | 0.96 | 929 |
|  | 505^†^ | Bay 2 - process operator | 90 | 41 | 1,264 | 171 | 5.07 | 2 | 62,260 |
|  | 506 | Bay 2 - process coordinator | 13 | 4 | 178 | 31 | 33.3 | 0.01 | 1,000 |
|  | 508 | Bay 3 - process operator | 72 | 35 | 724 | 90 | 7.57 | 0.02 | 18,910 |
|  | 509 | Bay 3 - process coordinator | 8 | 0 | 486 | 79 | 5.42 | 16 | 3,470 |
|  | 511 | Bay 4 (any job title) | 6 | 5 | 5 | 1.25 | 4.76 | 0.33 | 25 |
|  | 512^†^ | Bay 5 - process operator | 117 | 38 | 3,927 | 304 | 9.54 | 0.03 | 123,433 |
|  | 513 | Bay 5 - process coordinator | 11 | 2 | 1,945 | 59 | 12.8 | 3 | 19,600 |
|  | 514 | Bay 5 - brickmaker | 0 |  |  |  |  |  |  |
|  | 515 | Bay 5 - other job title | 2 | 2 | 1.21 | 0.94 | 2.87 | 0.44 | 2 |
| *Continued on next page* | | | | | | | | | |

**Table S1.** Continued from previous page

| **Facility** | | **Exposure Code** | | **Job title** | | **N** | | **ND** | | **AM** | **GM** | **GSD** | **Min** | **Max** |
| --- | --- | --- | --- | --- | --- | --- | --- | --- | --- | --- | --- | --- | --- | --- |
| 5 | 516 | | IM Building/Rhodium Sidestream - process operator | | 74 | | 41 | | 86 | | 6 | 10.3 | 0.01 | 2,000 |
|  | 517 | | IM Building/Rhodium Sidestream - process coordinator | | 9 | | 6 | | 35 | | 0.48 | 47.0 | 0.01 | 284 |
|  | 518 | | IM Building/Rhodium Sidestream - other job title | | 0 | |  | |  | |  |  |  |  |
|  | 519 | | Level 3/melting department - process operator | | 31 | | 18 | | 89 | | 33 | 6.35 | 0.01 | 970 |
|  | 520 | | Level 3/melting department - process coordinator | | 7 | | 3 | | 9 | | 3 | 13.4 | 0.01 | 18 |
|  | 522 | | Metal Support (any job title) | | 0 | |  | |  | |  |  |  |  |
|  | 523 | | Residue handling 436 - process operator | | 61 | | 32 | | 1,039 | | 130 | 9.38 | 0.02 | 12,400 |
|  | 524 | | Residue handling 436 - process coordinator | | 3 | | 3 | | 2 | | 2 | 1.35 | 1.38 | 2 |
|  | 525 | | Residue handling 436 - other job title | | 5 | | 3 | | 673 | | 324 | 3.39 | 143 | 2,500 |
|  | 526 | | Solvex - process operator | | 16 | | 8 | | 16 | | 9 | 3.33 | 1.36 | 59 |
|  | 527 | | Solvex - process coordinator | | 1 | | 0 | |  | |  |  | 15 | 15 |
|  | 528 | | Solvex - other job title | | 0 | |  | |  | |  |  |  |  |
|  | 529 | | Tank Farm (any job title) | | 7 | | 7 | | 1.05 | | 0.85 | 2.27 | 0.17 | 1.59 |
|  | 530 | | Utilities - process operator | | 9 | | 1 | | 37 | | 15 | 3.44 | 2 | 227 |
|  | 532 | | Utilities - other job title | | 1 | | 1 | |  | |  |  | 2 | 2 |
|  | 533 | | VRP - process operator | | 54 | | 32 | | 172 | | 12 | 12.2 | 0.02 | 2,000 |
|  | 534 | | VRP - process coordinator | | 15 | | 6 | | 15 | | 3 | 17.9 | 0.02 | 41 |
|  | 535 | | VRP - other job title | | 1 | | 1 | |  | |  |  | 0.01 | 0.01 |
|  | 536 | | Maintenance workers - Met Support | | 0 | |  | |  | |  |  |  |  |
|  | 537 | | Other maintenance workers | | 62 | | 19 | | 115 | | 28 | 5.61 | 1.14 | 1,720 |
|  | 538 | | Laboratory workers | | 77 | | 52 | | 68 | | 17 | 5.46 | 0.52 | 1,500 |
|  | 539 | | Other non-production workers occasionally going into the plant | | 2 | | 2 | | 80 | | 75 | 1.66 | 52 | 107 |
|  | 540 | | Other non-production workers never going into the plant | | 9 | | 9 | | 0.61 | | 0.34 | 3.55 | 0.06 | 2 |
|  | 541 | | Other | | 24 | | 7 | | 21 | | 11 | 4.00 | 0.77 | 132 |

N, number of measurements; ND, number of non-detectable measurements; AM, arithmetic mean (ng/m^3^); GM, geometric mean (ng/m^3^); GSD, geometric standard deviation; Min, minimum concentration (ng/m^3^); Max, maximum concentration (ng/m^3^); R&D, research and development; VRP, value recovery plant. All exposure codes which were present in the exposure and/or epidemiological data are listed here. AM and GM were calculated for exposure codes with two or more measurements. ^†^Exposure codes with time trends in estimated GM exposure levels that do not track with the facility-wide trends. ^*^Exposure code 501-503 will be excluded from subsequent exposure-response analysis because there is no involvement of chlorine here and therefore no potential for generation of chloroplatinates.

**Table S2.** Overview of the exposure groups and their exposure codes

| **Exposure group** | **Exposure codes** | | | | | | | |
| --- | --- | --- | --- | --- | --- | --- | --- | --- |
| **1** | 501^*^ | 503^*^ |  |  |  |  |  |  |
| **2** | 101 | 102 | 103 | 106 | 201 | 204 | 206 | 207 |
|  | 209 | 301 | 408 | 409 | 411 | 414 | 502^*^ | 505 |
|  | 512 | 514 |  |  |  |  |  |  |
| **3.1** | 104 | 105 | 107 | 108 | 205 | 208 | 302 | 303 |
|  | 304 | 305 | 306 | 307 | 310 | 311 | 313 | 314 |
|  | 317 | 319 | 401 | 402 | 403 | 404 | 405 | 406 |
|  | 407 | 410 | 412 | 413 | 415 | 416 | 417 | 418 |
|  | 419 | 420 | 421 | 422 | 423 | 429 | 506 | 508 |
|  | 509 | 513 | 515 | 519 | 520 | 523 | 524 | 525 |
| **3.2** | 110 | 203 | 312 | 425 | 427 | 428 | 526 | 527 |
|  | 530 | 532 | 533 | 534 | 535 | 538 |  |  |
| **4** | 109 | 202 | 308 | 309 | 431 | 432 | 434 | 435 |
|  | 437 | 537 | 541 |  |  |  |  |  |
| **5** | 211 | 315 | 424 | 426 | 438 | 439 | 440 | 441 |
|  | 442 | 528 | 539 |  |  |  |  |  |
| **6** | 444 | 504 | 511 | 516 | 517 | 518 | 522 | 529 |
|  | 536 |  |  |  |  |  |  |  |
| **7** | 111 | 210 | 318 | 540 |  |  |  |  |
| **No group** | 112 | 541 |  |  |  |  |  |  |

All exposure codes which were present in the exposure and/or epidemiological data are listed here. ^*^Exposure code 501-503 will be excluded from subsequent exposure-response analysis because there is no involvement of chlorine here and therefore no potential for generation of chloroplatinates.

- **Group 1:** Continuous exposure during regular work shifts; geometric mean >1000 ng/m^3^
- **Group 2:** Continuous exposure during regular work shifts; geometric mean 100-1000 ng/m^3^
- **Group 3.1:** Continuous exposure during regular work shifts; geometric mean <100 ng/m^3^ (workers in this group are all production workers with likely soluble Pt salt exposure in regular Pt production areas)
- **Group 3.2:** Continuous exposure during regular work shifts; geometric mean <100 ng/m^3^ (workers in this group have been separated from group 3.1 as they were not working in regular Pt production areas. Either they are laboratory workers or they work in production areas with low Pt salt exposure potential but still Pt areas)
- **Group 4:** Variable exposure, mainly maintenance workers
- **Group 5:** Occasional exposure (not full-time in the plant but occasionally entering the production area as part of their job)
- **Group 6:** Production workers in non-Pt areas
- **Group 7:** Non-exposed (full office-workers; non-production workers never going into the plant)
- **No group:** Exposure codes that did not fit in any of the groups above and were also not similar to each other (no relevant information available for grouping).

**Table S3.** Total number of personal soluble Pt salts measurements per year and facility between 2000 and 2016. Between parentheses, the number of measurements with levels below the limit of detection is indicated.

| **Year** | **Facility 1** | **Facility 2** | **Facility 3** | **Facility 4** | **Facility 5** | **Total (%ND)** |
| --- | --- | --- | --- | --- | --- | --- |
| **2000** | 0 (0) | 3 (3) | 47 (21) | 0 (0) | 0 (0) | 50 (48%) |
| **2001** | 0 (0) | 0 (0) | 29 (11) | 5 (4) | 0 (0) | 34 (44%) |
| **2002** | 0 (0) | 4 (3) | 70 (42) | 42 (21) | 0 (0) | 116 (57%) |
| **2003** | 0 (0) | 2 (0) | 63 (35) | 21 (11) | 63 (49) | 149 (64%) |
| **2004** | 8 (3) | 10 (8) | 0 (0) | 34 (27) | 42 (34) | 94 (77%) |
| **2005** | 15 (6) | 3 (3) | 9 (5) | 44 (43) | 16 (13) | 87 (80%) |
| **2006** | 29 (14) | 0 (0) | 45 (41) | 29 (27) | 63 (52) | 166 (81%) |
| **2007** | 42 (17) | 9 (7) | 13 (9) | 20 (16) | 59 (55) | 143 (73%) |
| **2008** | 27 (15) | 5 (4) | 0 (0) | 18 (14) | 36 (18) | 86 (59%) |
| **2009** | 48 (10) | 57 (18) | 46 (35) | 22 (17) | 54 (23) | 227 (45%) |
| **2010** | 22 (2) | 15 (1) | 61(54) | 169 (21) | 129 (22) | 396 (25%) |
| **2011** | 12 (1) | 17 (1) | 58 (47) | 75 (63) | 15 (15) | 177 (72%) |
| **2012** | 1 (0) | 9 (4) | 59 (34) | 83 (15) | 169 (28) | 321 (25%) |
| **2013** | 55 (5) | 38 (1) | 59 (26) | 60 (2) | 89 (9) | 301 (14%) |
| **2014** | 38 (1) | 87 (6) | 13 (12) | 53 (2) | 42 (42) | 233 (27%) |
| **2015** | 56 (6) | 59 (1) | 39 (10) | 54 (3) | 104 (30) | 312 (16%) |
| **2016** | 39 (9) | 51 (2) | 0 (0) | 0 (0) | 0 (0) | 90 (12%) |
| **Total (%ND)** | 392 (23%) | 369 (17%) | 611 (63%) | 729 (39%) | 881 (44%) | 2,982 (41%) |

% ND: Percentage of non-detectable measurements.

**Table S4.** Overview of personal exposure measurements of soluble Pt salts (ng/m^3^) for each combination of facility and job title (exposure codes) during the first part of the study (between 2000 and 2010) and during the second part of the study (between 2011 and 2016).

|  |  | **2000-2010** | | | |  | **2011-2016** | | | |
| --- | --- | --- | --- | --- | --- | --- | --- | --- | --- | --- |
| **Facility** | **Exposure Code** | **N** | **ND** | **GM** | **GSD** |  | **N** | **ND** | **GM** | **GSD** |
| 1 | 101 | 8 | 4 | 295 | 5.56 |  | 0 |  |  |  |
|  | 102 | 52 | 11 | 394 | 5.46 |  | 26 | 0 | 365 | 3.26 |
|  | 103 | 0 |  |  |  |  | 16 | 0 | 500 | 6.05 |
|  | 104 | 7 | 5 | 111 | 8.75 |  | 14 | 3 | 65 | 5.01 |
|  | 105 | 59 | 29 | 129 | 5.57 |  | 66 | 7 | 137 | 5.48 |
|  | 106^†^ | 52 | 12 | 253 | 3.66 |  | 32 | 0 | 393 | 2.67 |
|  | 107 | 5 | 0 | 117 | 3.05 |  | 3 | 0 | 121 | 2.76 |
|  | 108 | 7 | 5 | 48 | 3.54 |  | 14 | 3 | 34 | 2.59 |
|  | 109 | 1 | 1 | 47 |  |  | 11 | 4 | 23 | 2.81 |
|  | 111 | 0 |  |  |  |  | 6 | 5 | 2 | 2.62 |
|  | 112^†^ | 0 |  |  |  |  | 13 | 0 | 411 | 2.26 |
|  |  |  |  |  |  |  |  |  |  |  |
| 2 | 201 | 69 | 24 | 288 | 4.04 |  | 186 | 4 | 360 | 4.82 |
|  | 202 | 1 | 1 | 34 |  |  | 38 | 4 | 133 | 5.11 |
|  | 203 | 9 | 8 | 15 | 3.85 |  | 11 | 6 | 11 | 2.30 |
|  | 204 | 3 | 1 | 255 | 2.67 |  | 8 | 0 | 279 | 2.45 |
|  | 205 | 4 | 3 | 108 | 1.53 |  | 11 | 0 | 126 | 5.20 |
|  | 206 | 15 | 8 | 1,221 | 17.5 |  | 1 | 0 | 163 |  |
|  | 207 | 5 | 0 | 64 | 3.88 |  | 4 | 0 | 243 | 2.91 |
|  | 208 | 2 | 2 | 23 | 2.23 |  | 2 | 1 | 26 | 9.11 |
|  |  |  |  |  |  |  |  |  |  |  |
| 3 | 301 | 18 | 9 | 478 | 4.02 |  | 10 | 2 | 535 | 5.27 |
|  | 302 | 36 | 20 | 244 | 3.16 |  | 22 | 10 | 108 | 3.63 |
|  | 303 | 54 | 27 | 278 | 2.95 |  | 37 | 23 | 103 | 2.32 |
|  | 304 | 14 | 11 | 165 | 3.55 |  | 11 | 6 | 53 | 3.27 |
|  | 305^†^ | 44 | 38 | 94 | 2.37 |  | 18 | 7 | 188 | 5.40 |
|  | 306 | 67 | 54 | 137 | 2.96 |  | 15 | 9 | 96 | 3.81 |
|  | 307 | 19 | 13 | 195 | 3.08 |  | 13 | 7 | 58 | 2.48 |
|  | 308 | 52 | 33 | 190 | 3.25 |  | 35 | 22 | 103 | 3.05 |
|  | 309 | 10 | 6 | 288 | 5.78 |  | 14 | 7 | 155 | 2.79 |
|  | 310 | 19 | 14 | 146 | 2.43 |  | 17 | 13 | 59 | 2.78 |
|  | 311 | 8 | 5 | 210 | 2.90 |  | 12 | 7 | 129 | 1.91 |
|  | 312 | 17 | 12 | 150 | 2.94 |  | 1 | 1 | 94 |  |
|  | 313 | 5 | 2 | 542 | 4.97 |  | 1 | 1 | 44 |  |
|  | 314 | 1 | 0 | 1,336 |  |  | 0 |  |  |  |
|  | 315 | 2 | 2 | 59 | 1.49 |  | 0 |  |  |  |
|  | 317 | 1 | 0 | 3,636 |  |  | 4 | 3 | 58 | 1.86 |
|  | 318 | 8 | 5 | 142 | 2.34 |  | 0 |  |  |  |
|  | 319 | 8 | 2 | 1,118 | 4.65 |  | 18 | 11 | 60 | 2.37 |
|  |  |  |  |  |  |  |  |  |  |  |
| 4 | 401 | 16 | 10 | 42 | 3.27 |  | 2 | 1 | 4 | 8.96 |
|  | 402 | 12 | 6 | 43 | 3.49 |  | 3 | 0 | 44 | 1.98 |
|  | 403 | 19 | 11 | 52 | 2.83 |  | 3 | 0 | 49 | 1.23 |
|  | 404 | 17 | 10 | 52 | 3.38 |  | 3 | 0 | 52 | 4.57 |
|  | 405 | 31 | 20 | 40 | 3.58 |  | 1 | 0 | 80 |  |
|  | 406 | 32 | 20 | 68 | 3.73 |  | 3 | 0 | 77 | 6.87 |
|  | 408 | 13 | 3 | 348 | 3.96 |  | 30 | 8 | 69 | 15.9 |
|  | 409^†^ | 8 | 2 | 302 | 5.84 |  | 14 | 8 | 12 | 23.6 |
|  | 410 | 23 | 7 | 253 | 5.36 |  | 45 | 10 | 92 | 15.9 |
|  | 411 | 14 | 4 | 188 | 3.30 |  | 2 | 0 | 89 | 1.17 |
|  | 412 | 32 | 22 | 95 | 4.18 |  | 29 | 5 | 58 | 10.3 |
|  | 413 | 27 | 17 | 93 | 4.21 |  | 16 | 3 | 85 | 13.3 |
|  | 414^†^ | 16 | 3 | 443 | 7.70 |  | 13 | 3 | 45 | 12.1 |
|  | 415^†^ | 40 | 19 | 180 | 4.95 |  | 35 | 7 | 48 | 11.0 |
|  | 416 | 20 | 13 | 57 | 3.19 |  | 20 | 4 | 23 | 8.38 |
|  | 417^†^ | 10 | 7 | 84 | 5.04 |  | 9 | 2 | 27 | 9.16 |
|  | 418^†^ | 1 | 0 | 298 |  |  | 4 | 2 | 7 | 17.3 |
|  | 419 | 9 | 3 | 52 | 1.76 |  | 11 | 5 | 9 | 13.3 |
|  | 420 | 12 | 4 | 116 | 4.56 |  | 22 | 10 | 12 | 15.1 |
|  | 421 | 9 | 6 | 145 | 9.03 |  | 6 | 0 | 142 | 2.79 |
|  | 422 | 1 | 1 | 36 |  |  | 2 | 1 | 12 | 51.5 |
|  | 423 | 0 |  |  |  |  | 1 | 1 | 0.61 |  |
| *Continued on next page* | | | | | | | | | | |

**Table S4.** Continued from previous page

|  |  | **2000-2010** | | | |  | **2011-2016** | | | |
| --- | --- | --- | --- | --- | --- | --- | --- | --- | --- | --- |
| **Facility** | **Exposure Code** | **N** | **ND** | **GM** | **GSD** |  | **N** | **ND** | **GM** | **GSD** |
| 4 | 424 | 4 | 1 | 17 | 3.12 |  | 0 |  |  |  |
|  | 425 | 12 | 6 | 17 | 4.31 |  | 0 |  |  |  |
|  | 426 | 8 | 6 | 41 | 4.01 |  | 17 | 5 | 35 | 15.3 |
|  | 427 | 6 | 0 | 9 | 1.63 |  | 27 | 5 | 5 | 5.00 |
|  | 428 | 0 |  |  |  |  | 2 | 1 | 3 | 14.2 |
|  | 429 | 6 | 0 | 63 | 3.31 |  | 0 |  |  |  |
|  | 431 | 6 | 0 | 208 | 2.89 |  | 4 | 3 | 2 | 5.79 |
|  | 435 | 0 |  |  |  |  | 1 | 1 | 0.59 |  |
|  |  |  |  |  |  |  |  |  |  |  |
| 5 | 501^*^ | 22 | 3 | 4,349 | 8.17 |  | 41 | 1 | 331 | 4.76 |
|  | 502^*^ | 9 | 2 | 1,064 | 12.9 |  | 13 | 1 | 163 | 5.57 |
|  | 503^*^ | 0 |  |  |  |  | 1 | 0 | 2,450 |  |
|  | 504 | 0 |  |  |  |  | 8 | 5 | 5 | 11.0 |
|  | 505^†^ | 59 | 39 | 186 | 5.16 |  | 31 | 2 | 146 | 4.96 |
|  | 506 | 9 | 4 | 24 | 69.9 |  | 4 | 0 | 57 | 1.78 |
|  | 508 | 46 | 30 | 110 | 7.69 |  | 26 | 5 | 62 | 7.24 |
|  | 509 | 6 | 0 | 97 | 7.00 |  | 2 | 0 | 42 | 1.16 |
|  | 511 | 0 |  |  |  |  | 6 | 5 | 1.25 | 4.76 |
|  | 512^†^ | 71 | 35 | 416 | 12.2 |  | 46 | 3 | 187 | 5.58 |
|  | 513 | 7 | 1 | 53 | 6.20 |  | 4 | 1 | 73 | 47.3 |
|  | 515 | 0 |  |  |  |  | 2 | 2 | 1 | 2.87 |
|  | 516 | 27 | 17 | 14 | 27.9 |  | 47 | 24 | 4 | 3.69 |
|  | 517 | 6 | 4 | 0.2 | 84.5 |  | 3 | 2 | 3 | 4.64 |
|  | 519 | 31 | 18 | 33 | 6.35 |  | 0 |  |  |  |
|  | 520 | 5 | 2 | 3 | 21.2 |  | 2 | 0 | 5 | 4.79 |
|  | 523 | 52 | 30 | 158 | 8.97 |  | 9 | 1 | 41 | 9.64 |
|  | 524 | 0 |  |  |  |  | 3 | 2 | 2 | 1.35 |
|  | 525 | 5 | 3 | 324 | 3.39 |  | 0 |  |  |  |
|  | 526 | 4 | 4 | 11 | 2.38 |  | 12 | 3 | 9 | 3.76 |
|  | 527 | 0 |  |  |  |  | 1 | 4 | 15 |  |
|  | 529 | 0 |  |  |  |  | 7 | 0 | 0.85 | 2.27 |
|  | 530 | 0 |  |  |  |  | 9 | 7 | 15 | 3.44 |
|  | 532 | 0 |  |  |  |  | 1 | 1 | 2 |  |
|  | 533 | 36 | 22 | 19 | 16.5 |  | 18 | 1 | 5 | 3.90 |
|  | 534 | 11 | 4 | 3 | 27.8 |  | 4 | 10 | 3 | 4.08 |
|  | 535 | 1 | 1 | 0.01 |  |  | 0 |  |  |  |
|  | 537 | 11 | 9 | 66 | 4.26 |  | 51 | 2 | 23 | 5.66 |
|  | 538 | 41 | 35 | 32 | 3.04 |  | 36 | 10 | 8 | 6.98 |
|  | 539 | 2 | 2 | 75 | 1.66 |  | 0 |  |  |  |
|  | 540 | 0 |  |  |  |  | 9 | 17 | 0.34 | 3.55 |
|  | 541 | 1 | 1 | 132 |  |  | 23 | 15 | 10 | 3.28 |

N, number of measurements; ND, number of non-detectable measurements; GM, geometric mean (ng/m^3^); GSD, geometric standard deviation. All exposure codes in Table S1 with one or more measurements are listed here. ^†^Exposure codes with time trends in estimated GM exposure levels that do not track with the facility-wide trends. ^*^Exposure code 501-503 will be excluded from subsequent exposure-response analysis because there is no involvement of chlorine here and therefore no potential for generation of chloroplatinates.
